# Metaproteomic portrait of the healthy human gut microbiota

**DOI:** 10.1038/s41522-024-00526-4

**Published:** 2024-06-28

**Authors:** Alessandro Tanca, Antonio Palomba, Giovanni Fiorito, Marcello Abbondio, Daniela Pagnozzi, Sergio Uzzau

**Affiliations:** 1 Department of Biomedical Sciences, University of Sassari, Sassari, Italy; 2 Porto Conte Ricerche, Science and Technology Park of Sardinia, Tramariglio, Alghero Italy; 3 Clinical Bioinformatic Unit, IRCCS Istituto Giannina Gaslini, Genoa, Italy; 4 Unit of Microbiology and Virology, University Hospital of Sassari, Sassari, Italy

**Keywords:** Microbiome, Symbiosis

## Abstract

Gut metaproteomics can provide direct evidence of microbial functions actively expressed in the colonic environments, contributing to clarify the role of the gut microbiota in human physiology. In this study, we re-analyzed 10 fecal metaproteomics datasets of healthy individuals from different continents and countries, with the aim of identifying stable and variable gut microbial functions and defining the contribution of specific bacterial taxa to the main metabolic pathways. The “core” metaproteome included 182 microbial functions and 83 pathways that were identified in all individuals analyzed. Several enzymes involved in glucose and pyruvate metabolism, along with glutamate dehydrogenase, acetate kinase, elongation factors G and Tu and DnaK, were the proteins with the lowest abundance variability in the cohorts under study. On the contrary, proteins involved in chemotaxis, response to stress and cell adhesion were among the most variable functions. Random-effect meta-analysis of correlation trends between taxa, functions and pathways revealed key ecological and molecular associations within the gut microbiota. The contribution of specific bacterial taxa to the main biological processes was also investigated, finding that *Faecalibacterium* is the most stable genus and the top contributor to anti-inflammatory butyrate production in the healthy gut microbiota. Active production of other mucosal immunomodulators facilitating host tolerance was observed, including *Roseburia* flagellin and lipopolysaccharide biosynthetic enzymes expressed by members of Bacteroidota. Our study provides a detailed picture of the healthy human gut microbiota, contributing to unveil its functional mechanisms and its relationship with nutrition, immunity, and environmental stressors.

## Introduction

Along the gastrointestinal tract, many diverse microbial communities colonize the different mucosal and luminal sites. These sites are characterized by specific conditions affecting the equilibrium between members of the microbial consortia and the surrounding environment. Within the microbial communities, each species regulates its own metabolic pathways to improve biomass growth and replication and to protect the cell against toxicity and stressful conditions. Notwithstanding the taxonomic heterogeneity of the human gut microbiota (GM), microbial adaptation to this ecological environment implies the existence and interindividual sharing of keystone microbial members and their biological functions. To this end, “core” elements of the human GM are defined as those taxa and/or their functions that are shared among individuals^1^. In the past decades, the genomic features of the human GM have been investigated at different levels, including 16 S rRNA gene amplicon and high-quality shotgun metagenome sequencing, revealing distinct differences in the relative representation of GM members at several taxonomic levels^2–6^. Metatranscriptomic studies revealed that GM transcriptional profiles are significantly more individualized than functional potential profiles provided by metagenomics, but less variable than GM taxonomic composition, and identified a “core” and a “variable” metatranscriptome, including genes universally or differentially transcribed over time and across participants, respectively^7,8^. Gut metaproteomics, in turn, enables the identification and quantification of protein functions (and related metabolic pathways) actively expressed by the GM and thus significantly contributing to human physiology^9^. Although this approach has increasingly been applied to studies investigating the relationship between the GM and human diseases^10–12^, there is still a lack of knowledge about the “core” colonic metaproteome of a healthy human population, i.e., all those protein functions which could be considered as the “baseline” of GM biology requirements in non-diseased individuals. Further, little is known about the contribution of specific taxa to metabolic pathways and/or fluxes in physiological conditions. Despite the universally acknowledged existence of active metabolic cross-feeding between members of the GM, the whole complexity of microbial interplays is still far from being solved. Growing attention has been paid to monitoring the production of specific metabolites by intestinal microbes, including short-chain fatty acids (SCFAs), being key regulators in healthy metabolism and in metabolic disorders^13^. Robust data on the role of each taxon in the different metabolic networks are thus keenly required.

In a pioneering metaproteomics study, our group analyzed the fecal metaproteome of 15 healthy subjects from a native and highly monitored Sardinian population. We investigated conserved and variable GM functions expressed in that cohort, also examining the taxon-specific contribution to metabolic pathways (including polysaccharide degradation, glycan transport, glycolysis and SCFA production)^14^. Here, we aim to validate and extend the previous observations by re-analyzing 10 fecal metaproteomics datasets, for a total of 134 healthy individuals from different continents and countries. Taxonomic and functional data were parsed to identify a “core” healthy gut metaproteome, along with more variable features. In addition, we found strong correlation trends between taxa, functions and pathways with important ecological and molecular implications. Finally, we investigated the contribution of specific microbial taxa to the main metabolic pathways and biological processes, with a focus on SCFA biosynthesis, tricarboxylic acid (TCA) cycle and quorum sensing.

## Results

### Datasets and general metrics

Following a review of the scientific literature and a search for publicly available fecal metaproteomics datasets containing healthy subjects, we re-analyzed here eight datasets matching the inclusion criteria (see Methods for details). These datasets spanned different sample preparation and mass spectrometry methods and a wide geographic distribution, including cohorts from Australia, Canada, China, Germany, Italy, Spain and USA. The previously published Italian study^14^ was extended with further five healthy subjects and two unpublished datasets (also matching the inclusion criteria) were finally added, for a total of 10 datasets (their characteristics are listed in Table 1) and 134 healthy human subjects.

**Table 1:** Characteristics of the datasets (re-)analyzed in this study

Dataset code	# subjects	Country	Stool PT	Sample prep	LC run (min)	Instrument model	FM	ProteomeXchange accession	Reference
D01	6	Spain	yes	ISD	265	Q-Exactive	HCD	PXD020786	^56^
D02	16	Australia	yes	ISD	90	Q-Exactive	HCD	PXD008870	^57^
D03	20	China	no	FASP	150	Fusion	HCD	IPX0002453001	^58^
D04	17	Germany	no	Gel	165	Elite	CID	PXD010371	^59^
D05	19	Germany	no	FASP	155	Elite	CID	PXD034175	^60^
D06	20	Italy	no	FASP	180	Velos	HCD	PXD005780^a^	^14^^a^
D07	8	USA	yes	ISD	265	Fusion	HCD	PXD022433	^61^
D08	12	Italy	no	FASP	180	Q-Exactive	HCD	PXD046818	unpublished
D09	10	Italy	no	FASP	310	Velos	HCD	PXD046818	unpublished
D10	6	Canada	yes	ISD	120	Q-Exactive	HCD	PXD015482	^62^

*CID* Collision-induced dissociation, *FASP* Filter-aided sample preparation, *FM* fragmentation mode, *HCD* Higher energy collisional dissociation, *ISD* In solution digestion, *LC* Liquid chromatography, *PT* Pretreatment. All instruments were Orbitrap mass spectrometers from Thermo Fisher Scientific.

^a^The original study (and related dataset) reported data from 15 subjects; further 5 subjects from the same cohort had been analyzed following the same protocol and their data have been included in this study (raw MS files have been deposited in ProteomeXchange along with those of datasets D08 and D09).

The same bioinformatic pipeline for peptide identification, quantification and annotation (see Methods for details) was applied to the 10 datasets. A mean of 40,585 peptides matching the human gut metagenome database were quantified per dataset (single dataset metrics are provided in Supplementary Data 1). After filtering out peptides not assigned to bacterial or archaeal taxa and merging the results from the 10 datasets, a total of 51 phyla, 421 genera, 2757 Kyoto Encyclopedia of Genes and Genomes (KEGG) orthology (KO) functions, 150 KEGG pathways, 4343 phylum-specific functions and 8861 genus-specific functions were detected (Supplementary Data 2).

### Abundance and variability of gut metaproteomic features

Our first aim was to get closer to a definition of the “core” human healthy gut metaproteome, identifying taxonomic and functional features consistently present with high abundance and frequency in the GM of healthy individuals. To this purpose, we aggregated normalized peptide abundances from the 10 datasets according to taxon (namely, phyla and genera), function, pathway and taxon-specific functional feature annotations (Supplementary Data 2). Also, to counterbalance high variability and possible batch effects between different datasets, we ranked features based on their relative abundance in each subject, and then considered the rank distribution among all subjects (Supplementary Data 3).

**Fig. 1:**
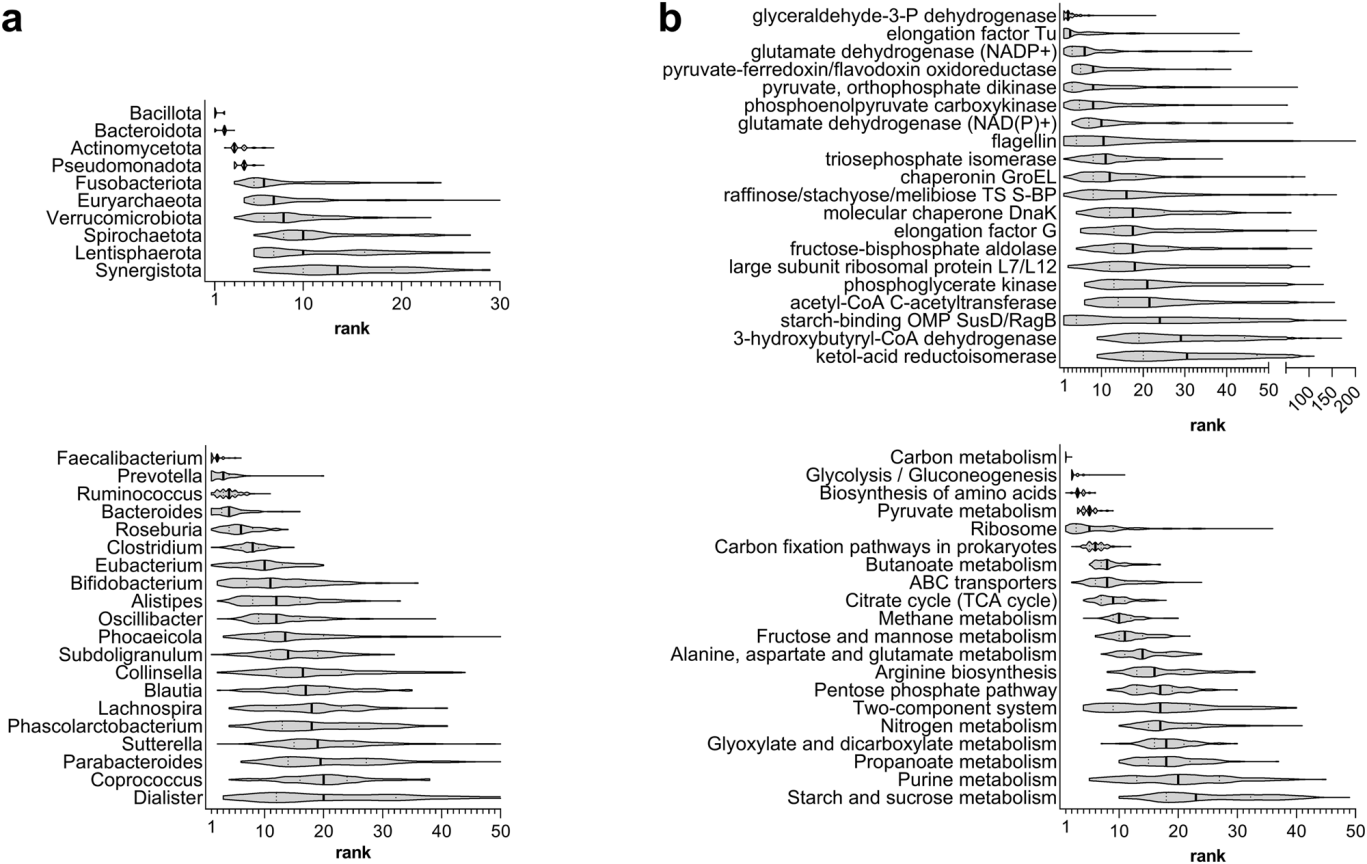
Main taxonomic and functional features of the healthy human gut metaproteome. Violin plots were based on the distribution of rank data, as observed in the 134 subjects analyzed in this study. Vertical black lines represent the median of the distribution. **a** Top 10 phyla (top) and top 20 genera (bottom), ordered by decreasing median rank. **b** Top 20 KO functions (top) and KEGG pathways (bottom), ordered by decreasing median rank. TS transport system; S-BP substrate-binding protein; OMP outer membrane protein.

In taxonomic terms, 4 phyla and 22 genera were detected in the gut metaproteome of all subjects, whereas 7 phyla and 45 genera were identified in all datasets. The most abundant taxa are shown in Fig. 1a (rank data) and Supplementary Fig. 1A (relative abundance data). Starting from higher taxonomic levels, Bacillota (formerly Firmicutes) was the first most abundant phylum in 78% of the subjects and the second in the remaining 22%, with an overall mean relative abundance of about 60%. Bacteroidota (formerly Bacteroidetes) was ranked as first, second and third phylum in 22%, 75%, and 3% of the subjects, respectively, with an overall mean relative abundance of about 35%. Actinomycetota (formerly Actinobacteria) and Pseudomonadota (formerly Proteobacteria) were at the third and fourth place (both with relative abundance around 2%). All other phyla were under 1% of abundance and much more variable in terms of median rank. Going down to the genus level, it must be noted that around 70% of the measured peptide abundance was related to peptides which could not be unambiguously assigned to a specific genus.

**Fig. 2:**
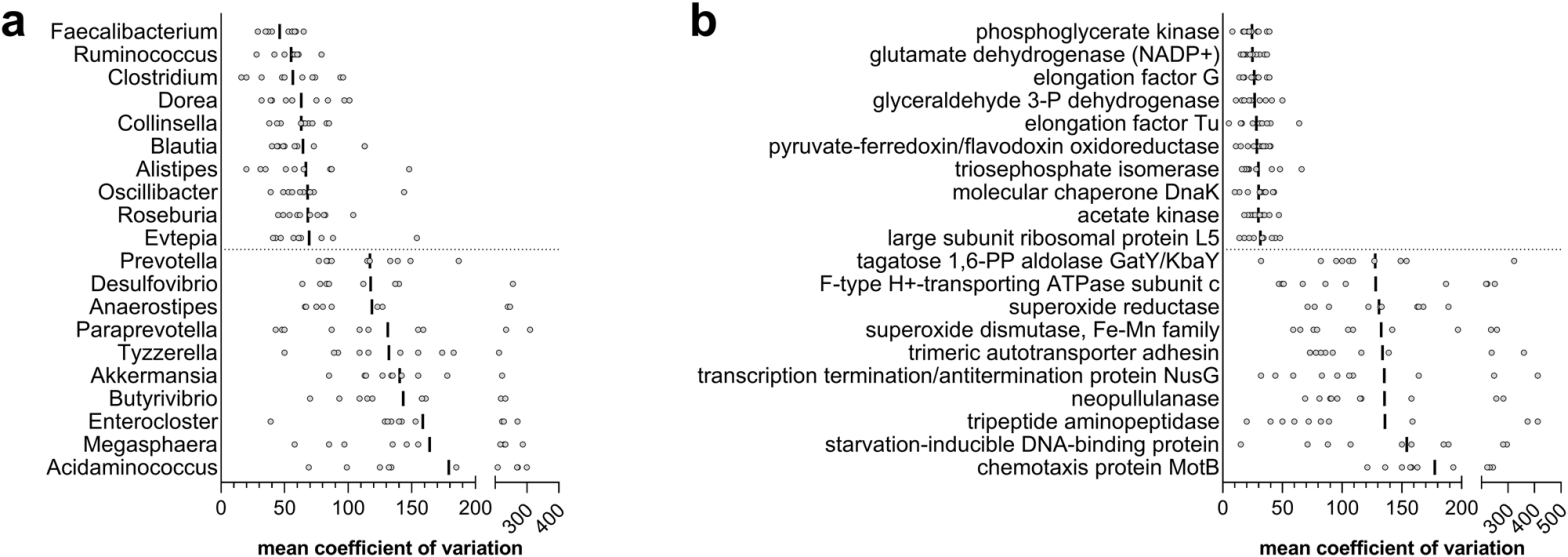
Variable and conserved features of the healthy human gut metaproteome. Aligned dot plots are based on the distribution of the mean coefficient of variation of the genus abundance among individuals, as observed in the 10 datasets analyzed in this study. Each grey dot represents a different dataset, while vertical black lines mark the mean of the distribution. Microbial genera (**a**) and KO functions (**b**) with the lowest (top 10) and highest (top 10) mean coefficient of variation values, among those detected in all datasets with > 0.01% mean abundance, are shown.

Considering the genus-specific peptides, *Prevotella* stands out as the genus with the higher mean relative abundance and the second higher rank, also showing a high variability between individuals, as demonstrated by the mean coefficient of variation (cv) data (Fig. 2a). On the contrary, *Faecalibacterium* was first in rank and second in relative abundance, but with the lowest cv among all genera. *Bacteroides* exhibited an intermediate cv value. Other 12 genera were among the top 20 based on rank data while exceeding 1% of mean relative abundance, including several Bacillota (*Ruminococcus*, *Roseburia*, *Eubacterium*, *Clostridium*, *Oscillibacter*, *Subdoligranulum* and *Dialister*, in addition to *Faecalibacterium*), a few Bacteroidota (*Bacteroides*, *Alistipes* and *Phocaeicola*, in addition to *Prevotella*) and two Actinomycetota (*Bifidobacterium* and *Collinsella*). Other Bacillota with lower median ranks, such as *Blautia*, *Dorea* and *Evtepia*, exhibited a very low variability between subjects in terms of mean abundance; on the contrary, very high cv values were observed for some genera, including *Acidaminococcus*, *Akkermansia*, *Anaerostipes*, *Butyrivibrio*, *Enterocloster*, *Megasphaera* and *Tyzzerella*.

In functional terms, 182 KO functions and 83 pathways were detected in the gut metaproteome of all subjects, whereas 422 KO functions and 115 pathways were identified in all datasets. The most abundant KO functions and pathways are shown in Fig. 1b (rank data) and Supplementary Fig. 1B (relative abundance data). Among the most abundant proteins we found many glycolytic enzymes, different types of glutamate dehydrogenase, several ABC transporters, chaperones and elongation factors, as well as flagellin and proteins belonging to the starch utilization system (Sus). The mean relative abundance of the two most represented proteins, glyceraldehyde 3-phosphate dehydrogenase (GAPDH) and elongation factor Tu, was around 5%. They also resulted among the functions with the lowest mean variability between subjects (Fig. 2b), along with less abundant proteins such as the terminal enzyme of acetogenesis, acetate kinase; on the contrary, the most variable functions were involved in many different processes, including chemotaxis, response to (oxidative) stress, cell adhesion and polysaccharide metabolism. Considering the most represented pathways, carbon metabolism (which includes enzymes belonging to many other carbohydrate metabolism pathways) was the best ranked pathway among all subjects, followed by glycolysis and amino acid biosynthesis. Other important metabolic pathways, such as pyruvate, butanoate (i.e., butyrate) and propanoate (i.e., propionate) metabolism, were among the top ranked, together with non-metabolic pathways as ribosome, ABC transporters and two-component system. As shown in Supplementary Fig. 1B, the mean abundance distribution of pathways appeared quite stable among subjects and datasets.

### Correlation trends between taxa, functions and metadata

We were also interested in investigating direct and inverse correlation trends between GM features, to identify co-occurrence and mutual exclusion dynamics. To this end, we calculated the Spearman’s coefficient of correlation (rho) for the abundances of the main taxonomic and functional features measured in the subjects (separately for each of the 10 datasets); then, we performed a random effect maximum likelihood (REML) meta-analysis to obtain a combined estimate for each feature pair (Supplementary Data 4).

First, we sought for correlations between taxa. At the phylum level (Supplementary Fig. 2), we observed as expected a significant inverse correlation between the two main phyla, Bacillota and Bacteroidota (rho = -0.85, FDR = 8.7 ∙ 10^−^^18^). At the genus level (Supplementary Fig. 3), the most significant results were the positive correlation between *Faecalibacterium* and *Lachnospira* (rho = 0.50, FDR = 1.4 ∙ 10^−^^5^) and the negative correlation between *Prevotella* and *Bacteroides* (rho = −0.48, FDR = 4.0 ∙ 10^−^^5^).

**Fig. 3:**
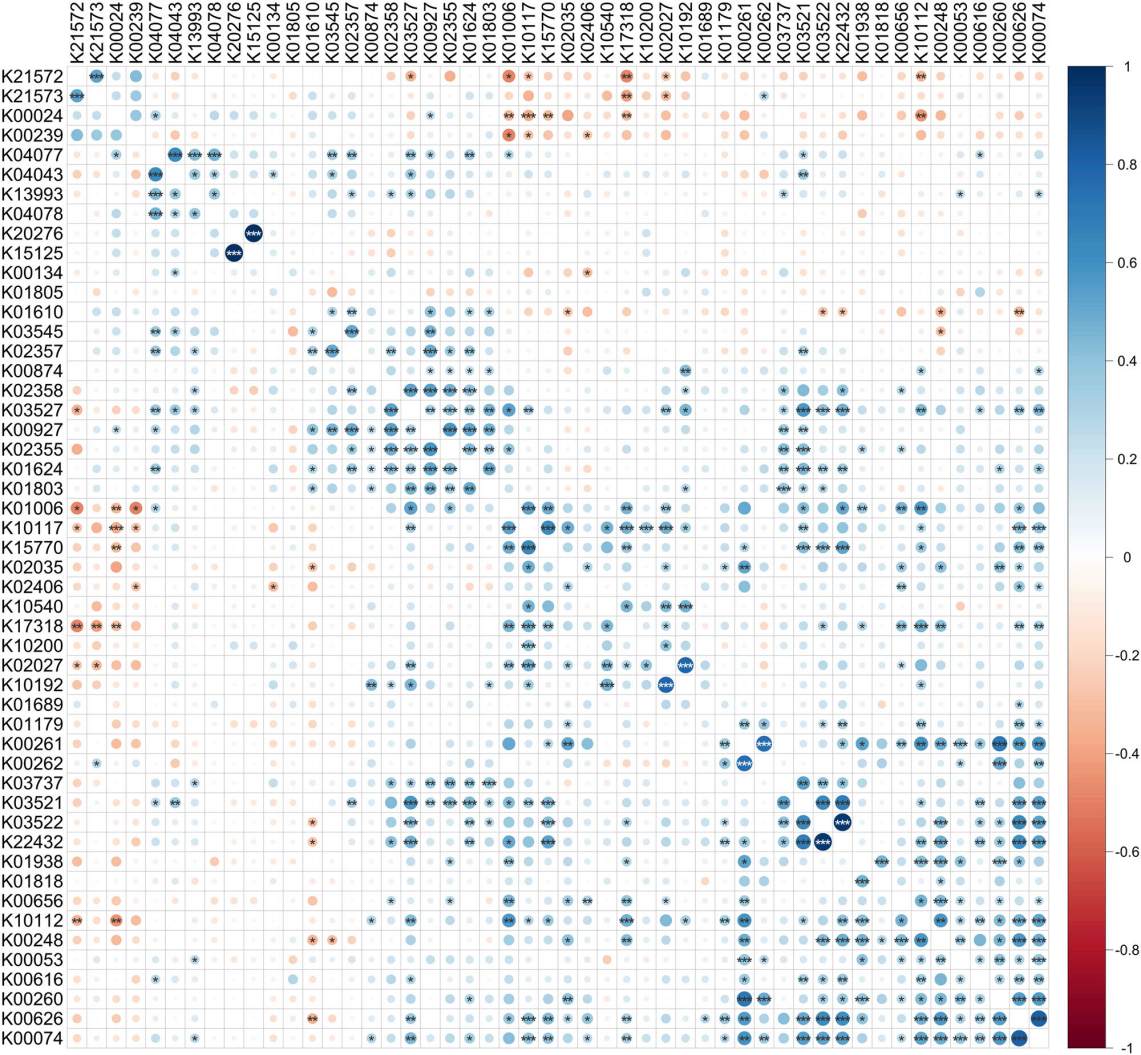
Heatmap showing correlation trends between KO functions according to Spearman’s rho values. The 50 KO functions with the highest relative abundance among those detected in all datasets and in at least 75% of subjects on average are shown (ribosomal proteins were not included). KO functions are ordered based on hierarchical clustering. Diameter and color of each circle (see legend on the right for color gradient) depend on the weighted average rho value computed via REML meta-analysis for that KO-KO correlation in the 10 datasets. Asterisks mark statistically significant correlations (*** = FDR < 0.001; ** = FDR < 0.01; * = FDR < 0.05). K21572, starch-binding outer membrane protein, SusD/RagB family; K21573, TonB-dependent starch-binding outer membrane protein SusC; K00024, malate dehydrogenase; K00239, succinate dehydrogenase flavoprotein subunit; K04077, chaperonin GroEL; K04043, molecular chaperone DnaK; K13993, HSP20 family protein; K04078, chaperonin GroES; K20276, large repetitive protein; K15125, filamentous hemagglutinin; K00134, glyceraldehyde 3-phosphate dehydrogenase (phosphorylating); K01805, xylose isomerase; K01610, phosphoenolpyruvate carboxykinase (ATP); K03545, trigger factor; K02357, elongation factor Ts; K00874, 2-dehydro-3-deoxygluconokinase; K02358, elongation factor Tu; K03527, 4-hydroxy-3-methylbut-2-en-1-yl diphosphate reductase; K00927, phosphoglycerate kinase; K02355, elongation factor G; K01624, fructose-bisphosphate aldolase, class II; K01803, triosephosphate isomerase (TIM); K01006, pyruvate, orthophosphate dikinase; K10117, raffinose/stachyose/melibiose transport system substrate-binding protein; K15770, arabinogalactan oligomer / maltooligosaccharide transport system substrate-binding protein; K02035, peptide/nickel transport system substrate-binding protein; K02406, flagellin; K10540, methyl-galactoside transport system substrate-binding protein; K17318, putative aldouronate transport system substrate-binding protein; K10200, N-acetylglucosamine transport system substrate-binding protein; K02027, multiple sugar transport system substrate-binding protein; K10192, oligogalacturonide transport system substrate-binding protein; K01689, enolase; K01179, endoglucanase; K00261, glutamate dehydrogenase (NAD(P) + ); K00262, glutamate dehydrogenase (NADP + ); K03737, pyruvate-ferredoxin/flavodoxin oxidoreductase; K03521, electron transfer flavoprotein beta subunit; K03522, electron transfer flavoprotein alpha subunit; K22432, caffeyl-CoA reductase-Etf complex subunit CarE; K01938, formate--tetrahydrofolate ligase; K01818, L-fucose/D-arabinose isomerase; K00656, formate C-acetyltransferase; K10112, multiple sugar transport system ATP-binding protein; K00248, butyryl-CoA dehydrogenase; K00053, ketol-acid reductoisomerase; K00616, transaldolase; K00260, glutamate dehydrogenase; K00626, acetyl-CoA C-acetyltransferase; K00074, 3-hydroxybutyryl-CoA dehydrogenase.

More interestingly, we found 248 significant correlations between the top 50 KO functions (Fig. 3). Among them, several correlation networks were identified, mainly involving enzymes with a role in pyruvate and butyrate metabolism (such as acetyl-CoA C-acetyltransferase, 3-hydroxybutyryl-CoA dehydrogenase and butyryl-CoA dehydrogenase), chaperones (such as GroEL, GroES, DnaK and HSP20) and ABC transporters (with various oligosaccharides and sugar acids as targets). These latter, in turn, showed inverse correlations with Sus members or enzymes as malate dehydrogenase. We also examined the top 50 most variable KO functions (based on cv values; Supplementary Fig. 4), finding strong direct correlations between enzymes involved in sulfur metabolism (mainly expressed by *Desulfovibrio*), as well as between several proteins (NADH-quinone oxidoreductase, chemotaxis protein MotB, DedD protein, amidophosphoribosyltransferase) dealing with very different biological activities (but all expressed mainly by Bacteroidota).

**Fig. 4:**
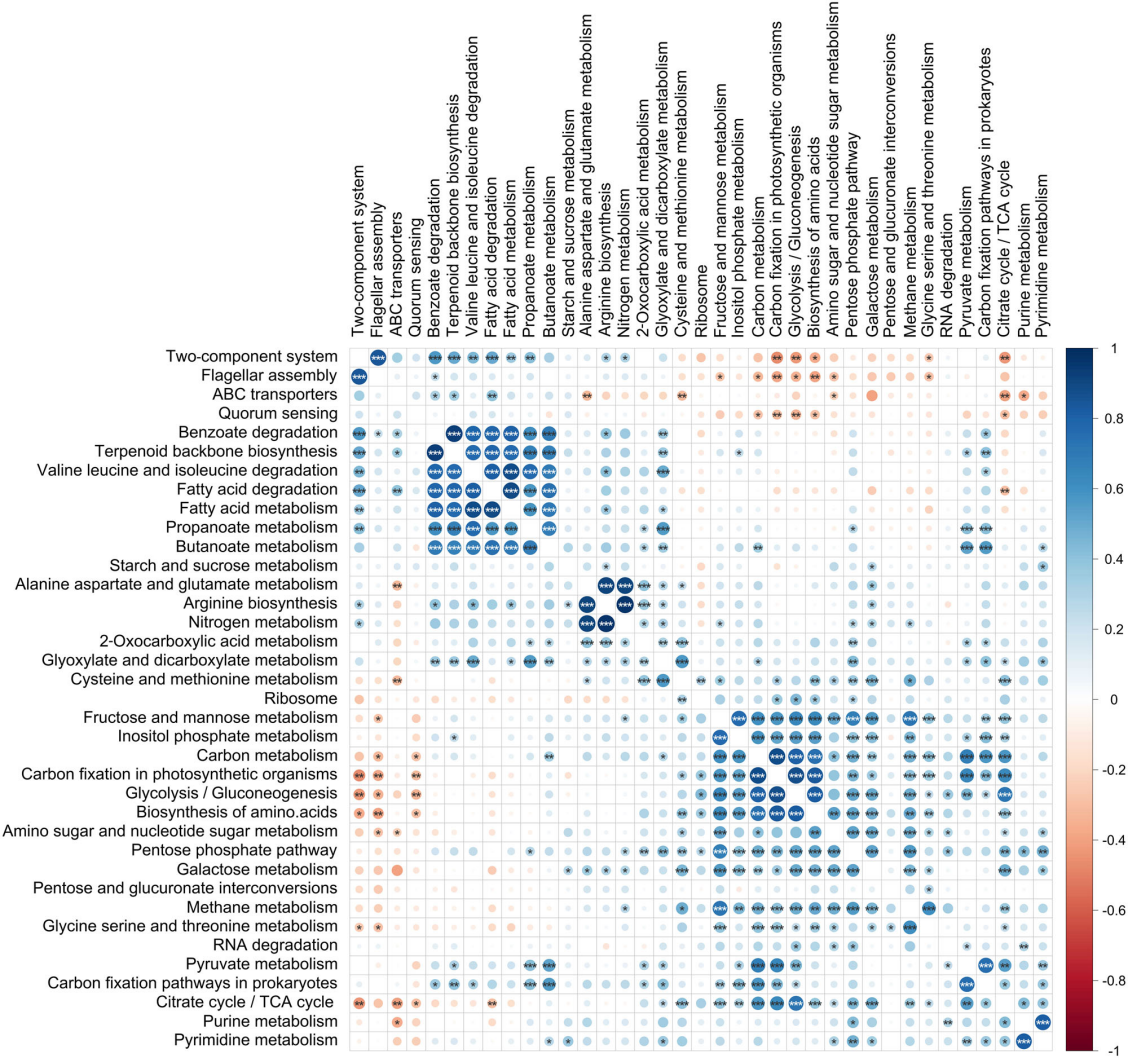
Heatmap showing correlation trends between KEGG pathways according to Spearman’s rho values. Pathways exceeding 1% of mean abundance and detected in all subjects are shown. Pathways are ordered based on hierarchical clustering. Diameter and color of each circle (see legend on the right for color gradient) depend on the weighted average rho value computed via REML meta-analysis for that pathway-pathway correlation in the 10 datasets. Asterisks mark statistically significant correlations (*** = FDR < 0.001; ** = FDR < 0.01; * = FDR < 0.05).

Moving to pathways (Fig. 4), 212 significant correlations could be detected, even with extremely high rho values (also due to the considerable number of enzymes shared between different pathways; see, for instance, fatty acid, butanoate, propanoate and benzoate metabolism pathways). More intriguingly, inverse correlations were identified between glycolysis and features with relevant biological implications such as quorum sensing and flagellar assembly.

We also investigated the correlation of the relative abundance of the main taxa and functions with subjects’ chronological age, using the same meta-analysis approach described above. However, no significant correlations could be found.

### Taxon-specific contribution to functions

Another key aim of this study was to investigate the taxon-specific contribution to functions and pathways actively expressed by the members of the human GM in physiological conditions. Accordingly, peptide abundance data were aggregated based on taxonomic and functional annotations, obtaining lists of phylum-specific and genus-specific functional features (namely, KEGG KO functions and pathways), along with their relative abundances and ranks (Supplementary Data 2 and 3). As shown in Supplementary Fig. 5A, most of the best ranked phylum-specific functions belonged to Bacillota, with starch-binding outer membrane protein (OMP) SusD/RagB being the best ranked function expressed by Bacteroidota. Down to lower taxonomic levels, most of the best ranked genus-specific functions were assigned to *Faecalibacterium* and included several ABC transporters and enzymes involved in glycolysis and butyrogenesis; of note, *Roseburia* flagellin was the second-best ranked protein, with Sus proteins from *Prevotella* and *Bacteroides* and cellulose 1,4-beta-cellobiosidase from *Ruminococcus* being as well among the top ranked functions. Moving to pathways (Supplementary Fig. 5B), Bacillota- and *Faecalibacterium-*specific pathways were by far the best ranked. According to these data, *Faecalibacterium* appeared to be mainly involved in carbohydrate transport and metabolism, especially in butyrate biosynthesis.

**Fig. 5:**
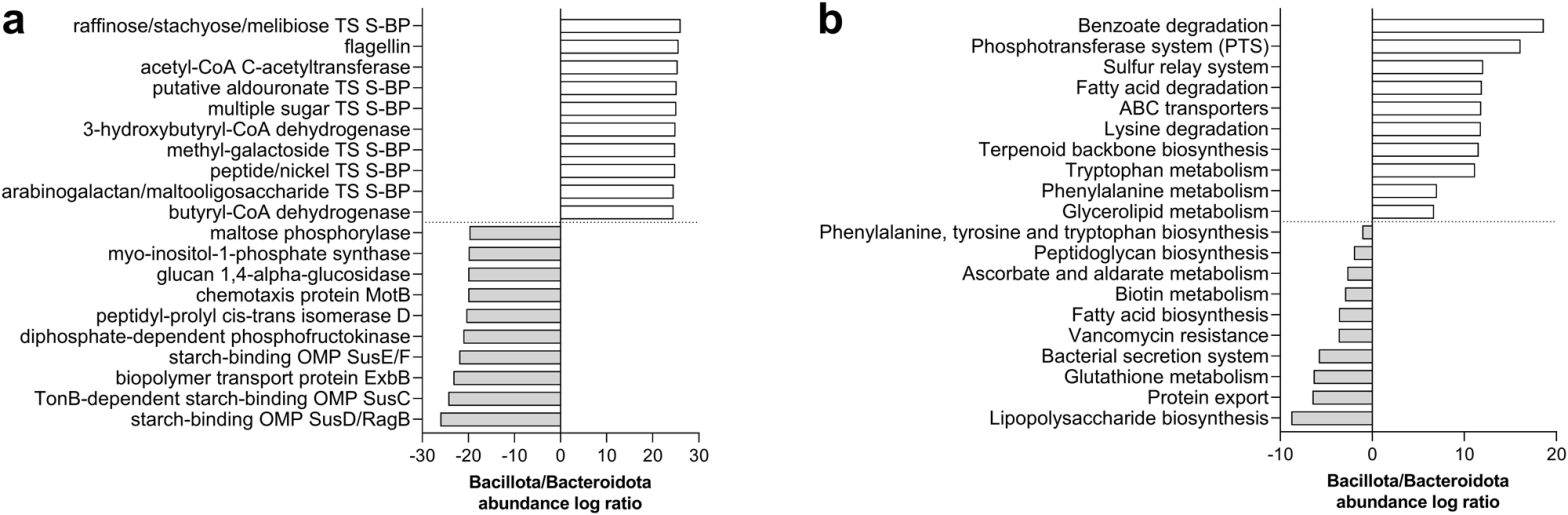
Functional features with significantly differential abundance between Bacillota and Bacteroidota. KO functions (**a**) and KEGG pathways (**b**) with the highest (top 10) and lowest (top 10) Bacillota/Bacteroidota abundance log ratios are shown. TS transport system, S-BP substrate-binding protein, OMP outer membrane protein.

Then, we specifically compared the metaproteome profile of the two main phyla, i.e., Bacillota and Bacteroidota, to find which functions and pathways could be identified as typically expressed by each of these phyla in the healthy GM (Supplementary Data 5). As shown in Fig. 5, several ABC transporters, butyrogenic enzymes and flagellin were typical of Bacillota (highest abundance log ratios), along with benzoate degradation, phosphotransferase system and sulfur relay system (among others) within pathways; on the other hand, several Sus members were among the functions most typically expressed by Bacteroidota, with LPS biosynthesis, protein export, and glutathione metabolism being the pathways with the lowest log ratios in the Bacillota vs Bacteroidota comparison.

Moreover, among functions with missing annotation at the genus level for < 50% of their abundance, we selected those assigned at ≥ 90% (on average) to a single genus to identify proper “genus-specific” functions. As a result, over 50 genus-specific protein functions were defined (Supplementary Data 5), including several enzymes exclusively expressed by *Prevotella*, *Faecalibacterium*, *Ruminococcus* and *Oscillibacter*, along with those responsible for methanogenesis and produced by the archaeon *Methanobrevibacter*. On the opposite direction, we also filtered functions to find those assigned at <75% (on average) to a single genus, i.e., expressed simultaneously by various members of the GM. These comprised 48 functions (Supplementary Data 5), including important enzymes such as alpha-amylase (expressed by *Ruminococcus*, *Eubacterium* and *Prevotella*), beta-glucosidase (*Prevotella* and *Bacteroides*) and dissimilatory sulfite reductase (*Bilophila* and *Desulfovibrio*).

### Role of gut microbiota members in SCFA biosynthesis, TCA cycle and quorum sensing

**Fig. 6:**
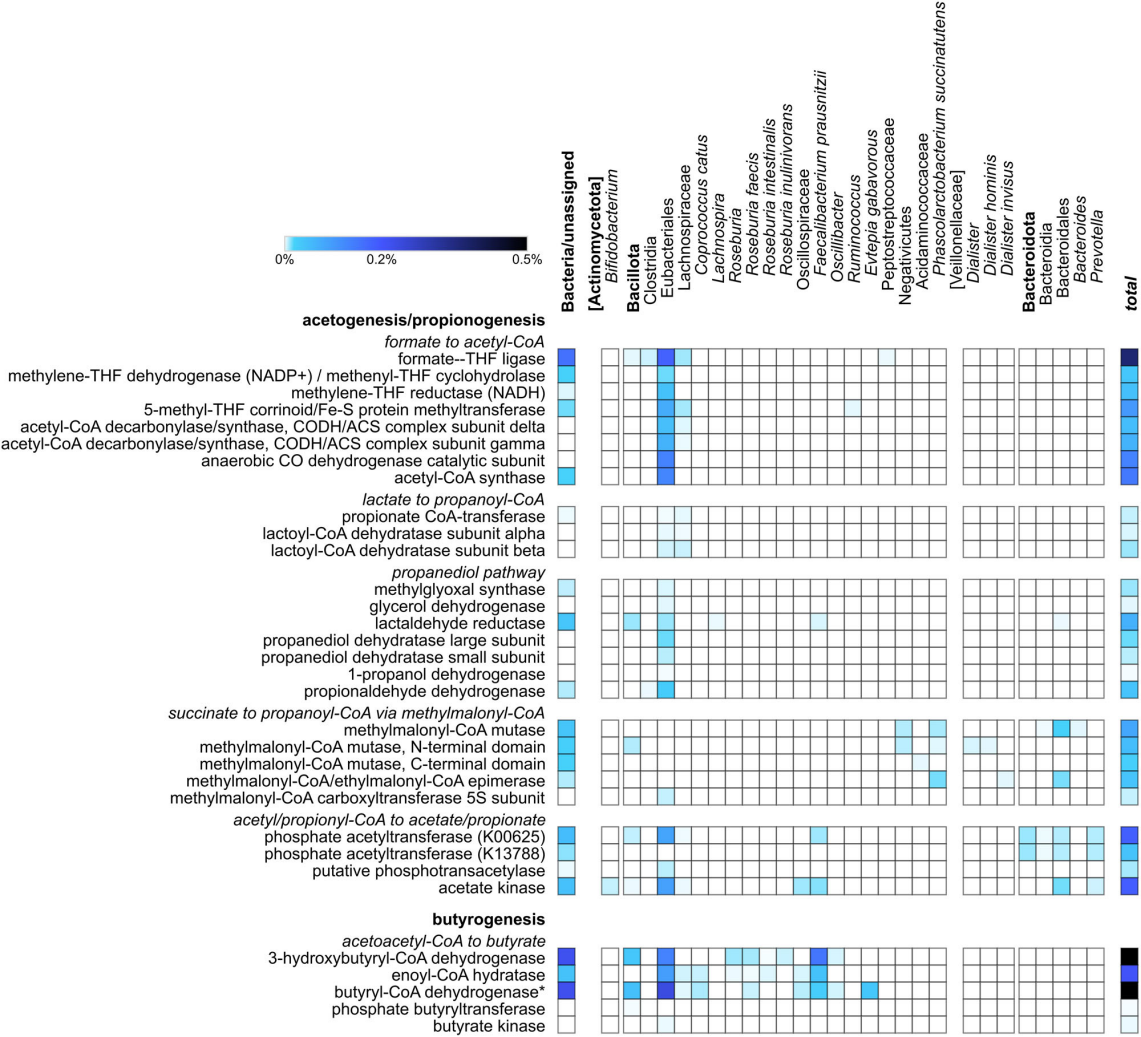
Taxon-specific distribution of enzymes involved in short-chain fatty acid (SCFA) biosynthesis. KO functions detected in more than 6 datasets, with a mean abundance higher than 0.001% and mapping to KEGG pathways named carbon fixation pathways in prokaryotes (Wood-Ljungdahl pathway part), propanoate metabolism and butanoate metabolism are reported. Enzymes are listed based on the sequential order of the reactions within the pathways; each sub-pathway is preceded by a subtitle in italic. The color of each square corresponds to the mean percentage abundance between the 10 datasets (see color legend). Phyla are in bold, genera and species in italic, while higher taxa useful for classification but unassigned to any of the listed enzymes are into square brackets. The column “total” corresponds to the summed abundance of all taxon-specific assignments (including “Bacteria/unassigned”) for a given enzyme. THF, tetrahydrofolate. Enzymes from other butyrogenic pathways (glutarate pathway, lysine pathway, 4-aminobutyrate/succinate pathway) were detected in a small number of datasets and therefore the only acetyl-CoA pathway was selected for butyrogenesis. Acetyl-CoA C-acetyltransferase, although highly expressed in all the datasets, was not specifically included within butyrogenesis as it participates in many other diverse pathways. * Butyryl-CoA dehydrogenase (K00248) can also catalyze propionyl-CoA biosynthesis from acryloyl-CoA (last step of “lactate to propanoyl-CoA” part of propionogenesis).

Finally, we focused on some pathways of interest to dissect the taxon-specific contribution to their metabolic steps and/or main components. Firstly, we selected the different pathways responsible for SCFA biosynthesis, namely the Wood-Ljungdahl acetogenic pathway^15^ (included within the KEGG pathway named carbon fixation pathways in prokaryotes), succinate, acrylate and propanediol propionogenic pathways^16^ (within the KEGG propanoate metabolism pathway) and the acetyl-CoA butyrogenic pathway^17^ (within the KEGG butanoate metabolism pathway), due both to their known relevance for host physiology and to their presence among the best ranked pathways in the analyzed datasets. Figure 6, based on the lowest common ancestor (LCA) taxonomic assignment of their peptides identified in the 10 datasets, illustrates the taxon-specific contribution to SCFA biosynthesis. Note that acetogenesis and propionogenesis are presented together as the enzymes involved in the two final steps of acetogenesis leading to acetate, according to the data from this study and to the information provided by the KEGG database, might also catalyze the last reactions of propionate production; other more specifically propionogenic enzymes capable to carry out the same reactions (e.g., propionate kinase) were found at very low abundances and/or in a small number of datasets.

As a result, enzymes catalyzing the acetogenic reactions from formate to acetyl-CoA were assigned mainly to Eubacteriales, but not to lower levels, most likely due to a high degree of conservation of their sequences among the members of this order. Similar considerations can be made about the propanediol pathway within propionogenesis; on the contrary, it can be observed how members of the Negativicutes, namely *Phascolarctobacterium succinatutens* and *Dialister* spp., are responsible, as expected, for most reactions of the succinate pathway (via methylmalonyl-CoA), together with Bacteriodales. Phosphate acetyltransferase and acetate kinase were both expressed by several members of Eubacteriales and Bacteroidales, including *Faecalibacterium* and *Prevotella*. Moving to butyrogenesis, despite a clearly high level of conservation of the enzyme sequences, some peptides could be consistently assigned down to the genus or species level, namely to *Roseburia*, *Faecalibacterium*, *Oscillibacter* and *Evtepia* spp.

We were also interested in identifying which types of microbes were specifically able to use the citrate cycle. As shown in Supplementary Fig. 6, the sequences of the enzymes involved in this pathway appear to be very conserved, as in most cases they could only be identified as bacterial, with no more specific taxonomic assignments. However, succinyl-CoA:acetate CoA-transferase could be assigned to *Phascolarctobacterium succinatutens* and *Dialister*, consistently with their known ability to metabolize succinate. Furthermore, other enzymes were expressed by Bacteroidales and their members (mainly *Prevotella*, but also *Bacteroides* and *Alistipes* when considering malate dehydrogenase). The contribution of Eubacteriales was instead minimal.

Finally, we focused our attention on proteins involved in the KEGG pathways flagellar assembly and quorum sensing, because of their quantitative and biological relevance. The abundance of the former pathway was essentially due to flagellin, which in turn was assigned at different taxonomic levels (from phylum to species), including genera such as *Roseburia*, *Eubacterium* and *Clostridium* (Supplementary Data 2). As far as quorum sensing is concerned (Supplementary Fig. 7), a heterogeneous group of peptide/protein transporters and translocases were identified. Transporters were mapped mainly to members of Bacillota (including *Faecalibacterium*, *Roseburia*, *Oscillibacter*, *Clostridium*, *Lachnospira*) and Actinomycetota (*Bifidobacterium* and *Collinsella*), whereas translocases were only assigned to members of Bacteroidota (mainly *Prevotella*).

## Discussion

Fecal metaproteomics allows the collection of functional data concerning the microbial consortia that colonize the distal colon. Even though the fecal microbiome is insufficient to represent the whole GM, the functions expressed by the colonic microbiota mirror the microbial adaptation to an ecosystem that is strongly affected by diet and health, and also conditioned by host secretions and microbial members coming from the upper traits. Further, its relative ease of access has enabled many large cohort studies by 16 S rDNA and shotgun metagenomics analyses. As a proxy for human GM, stool is the most frequently used sample also for metaproteomics studies. Therefore, to portrait the “core” metaproteome of healthy human GM we analyzed the currently available metaproteomic datasets from healthy human fecal samples. Due to high variability in sample processing and analytical methods between datasets (beyond genetic and environmental differences between cohorts/countries), all results were rank-based or expressed as mean between datasets.

Significant variability of the healthy GM composition at low taxonomic levels has been demonstrated by DNA sequencing^1^. We have previously detected similar variability integrating shotgun metagenomic and metaproteomic analyses^14^. At higher taxonomic levels, instead, high abundances of the phyla Bacillota and Bacteroidota are consistent between datasets and individuals, showing beyond doubt their overall relevance for the GM biology and interaction with the host. Both phyla include genera with variable interindividual distribution and with strikingly different functions (i.e., Bacillota *Clostridium* spp. vs *Lactobacillus* spp.). However, we also observed genera present in all individuals and with very low interindividual variability, a mark of keystone taxa highly connected with a healthy GM ecosystem. Our data showed *Faecalibacterium* as the most stable genus. As already assessed by metagenomic studies, this genus can account for up to 5% of the entire fecal microbiota in healthy individuals^18^. *Faecalibacterium* has been suggested to support intestinal health and it has been characterized by functional assays, showing anti-inflammatory properties due to its butyrate production and lack of features involved in epithelial cell adhesion and antimicrobial production^19^. In agreement with metagenomic data, we observed here that *Faecalibacterium* mean abundance was among 5.70% and 20.75% (when accounting or not for the abundance of peptides unassigned at the genus level) and stands as the top contributor to butyrate production in the healthy GM. Moreover, our analyses provide a list of features expressed by *Faecalibacterium* (as well as others expressed by other taxa) with very low interindividual variability in the healthy GM. These data can be further used to identify *Faecalibacterium* antigens/biomarker candidates to monitor the relative abundance of this taxon in case-control studies of clinical relevance. The only genus with abundance similar to that of *Faecalibacterium* was *Prevotella*. However, *Prevotella* showed a high variability between individuals according to metaproteomic data. Consistently with previous metagenomics reports^20^, we show that its abundance was inversely correlated to that of *Bacteroides*. Interestingly, both genera showed starch-binding OMP SusD/RagB as their top KO function. Their competition on the uptake and degradation of complex polysaccharides can be the foundation for the inverse correlation of their abundance values. As opposite to *Faecalibacterium*, *Prevotella* and its species (i.e., *P. copri* complex) were also described as heterogeneous in terms of genetic potential for metabolic functions, antimicrobial resistance, and correlations with the human immune response and health^21^. More recently, species belonging to this genus have been reassigned into seven genera whose distribution differ significantly between human populations, likely because of distinct dietary choices and exposure to antimicrobials^22^. Low-abundance genus *Evtepia* can also play a role as keystone member of the GM, since its stability was remarkable in all metaproteomic dataset analyzed. *Evtepia* most abundant KO was butyryl-CoA dehydrogenase, suggesting that production of SCFAs can be among the prevalent metabolic activity of this genus. On the contrary, the high variability of *Akkermansia* relative abundance (and the quite frequent lack of its detection) in a healthy cohort contrasts with its widely accepted importance for the GM homeostasis and casts doubts on its presence as an essential requisite to achieve healthy GM conditions, as recently indicated^23^.

The “core” metaproteome of the healthy human GM included 182 functions and 83 pathways that were detected in all 134 individuals analyzed in this study. The present re-analysis provides a definitive view of how certain microbial functions and pathways are evenly distributed in healthy subjects. As previously reported^14,24^, the most represented proteins are those involved in carbohydrate metabolism, energy production and translation. Other highly abundant functions are proteins typical of the predominant phyla Bacillota and Bacteroidota. Hence, their abundance also depends on the Bacillota/Bacteroidota ratio. Strikingly, these top functions represent GM elemental roles that are equivalent in the two phyla: uptake of carbohydrates and other energy sources (Bacillota phosphotransferase system and ABC transporters vs Bacteroidota Sus members), proper oxidation state of thiols (Bacillota sulfur relay system vs Bacteroidota glutathione metabolism) and production of active mucosal immunomodulators (Bacillota flagellin vs Bacteroidota LPS). These two last molecular classes represent Bacteroidota- and Bacillota-specific highly abundant functions/pathways due to obvious structural reasons. Flagellin and LPS are supramolecular structures with totally different functions on the bacterial surfaces. However, they are both also key for the dynamic relationship between the gut colonizing bacteria and the host immune response. Recent studies provided compelling evidence that most of the GM LPS, once considered among the main pro-inflammatory microbial associated molecular patterns (MAMPs) in the gut lumen, shows taxon-dependent effects on TLR4 signaling to cytokine expression and secretion. Specifically, TLR4 recognition of LPS encoded by Pseudomonadota members *Escherichia coli* and *Pseudomonas* spp., as described for many pathogenic bacteria, triggers intense inflammation^25^. On the contrary, Bacteroidota LPS has an immunoinhibitory activity, derived from an underacylated structural feature, silencing the TLR4 signaling by the other members of the GM^26^. Similarly, flagellin is a potent pro-inflammatory MAMP that binds to TLR5, contributing to the inflammation triggered by diverse bacterial pathogens belonging to Pseudomonadota. Here, we reported that this antigen is mostly encoded by Lachnospiraceae (*Roseburia*) in all healthy individuals of the 10 datasets. As for Bacteroidota LPS, *Roseburia* (and other Lachnospiraceae members including *Lachnospira*) produces an anti-inflammatory flagellin that does not activate TLR5 signaling^27,28^. Hence, despite their structural differences and taxonomic distributions, both Bacteroidota LPS and Bacillota flagellin appeared to be converging in their role of preserving from mucosal inflammation. Factors supporting the abundance of “silent” flagellins might be also relevant to sustain *Faecalibacterium* abundance and production of anti-inflammatory butyrate, since the strongest positive correlation that we observed at genus level was the one between *Faecalibacterium* and *Lachnospira*. The consistently high abundance of Bacteroidota and Bacillota, regardless their ratio, with their load of LPS and flagellins might then represent a key outcome of the GM-host co-evolution, ensuring steady protection from mucosal inflammation in a densely colonized gut.

As observed at the taxonomic level, several KO functions could be stably detected in all individuals of the cohorts analyzed with a low coefficient of variation. Ubiquitous distribution in healthy individuals and constitutive expression would be the ideal characteristics of “housekeeping” GM proteins. However, most of the ubiquitous functions measured in this study showed direct or inverse correlation with at least one another ubiquitous metabolic function, highlighting the specific connection with the microbial community biology and, therefore, disallowing their use as housekeeping proteins. Interestingly, GAPDH appeared consistently present in all individuals, at high abundance and low variation, and with no correlation with other – variable – GM metabolic functions. GAPDH is a moonlighting protein that functions as glycolytic enzyme as well as uracil DNA glycosylase and has been used for decades as a housekeeping protein in proteomic studies on eukaryotic cell and tissues, although its use as internal standard should be examined carefully in relation to the experimental conditions and disease state^29^. The relative abundance of the diverse GAPDH specifically encoded by the members of the human GM showed significant correlation with the respective taxon (data not shown). These data suggest the use of GAPDH as an internal reference for the abundance of its specific taxon. Further analyses are required to validate the human GM encoded GAPDH as a housekeeping protein to normalize variation of other functions in comparative case-control studies based on metaproteomic data and/or immunological assays.

In addition to the functions that are detected as stably present in all individuals, also those that are measured only in a small fraction of individuals and/or with an uneven distribution between individuals are of great interest. Within this category, we found enzymes that react with harmful oxidants and functions involved in bacterial cell adhesion and motility. Superoxide reductase and dismutase were already found among the most variable proteins in our previous study^14^. Other functions of interest are starvation inducible DNA binding protein, trimeric autotransporter adhesin, and chemotaxis protein MotB, the latter showing a strong pH-dependent expression, being affected by stress factors acting on the bacterial proton motive force^30^. These functions might then represent “accessory” proteins and pathways, responding to less common triggers, including transient nutritional factors, pH variations, oxidative and nitrosative stress^31^. Given their involvement in bacterial response to environmental stress, these moieties might then be monitored as signatures of subclinical events under healthy clinical conditions.

Another key driver of GM evolution is diet. Acetate, propionate, and butyrate are among the most abundant end products of GM fermentation of non-digestible carbohydrates, also representing the major flow of carbon from the GM to the host^32^. Our data provide direct evidence of the major contribution of Negativicutes (*Phascolarctobacterium* and *Dialister*) and Bacteroidales (*Bacteroides* and *Prevotella*) to propionate production via succinate. Succinate can be produced by the reversal of partial TCA cycle reactions, from fumarate reduction, as a primary cross-feeding metabolite between *Bacteroides* producers and *Phascolarctobacterium* utilizers^33^. While succinate production by *Bacteroides* and *Prevotella* is key for propionate production (via reductive TCA cycle), our taxon-specificity analysis of TCA cycle functions confirmed that most commensals, as obligate anaerobes, do not express (or express only partially) the enzymes of this pathway. An incomplete TCA cycle was clearly observed for Eubacteriales, including *Faecalibacterium*. Notably, this strictly anaerobe is associated both to the luminal and the mucosal GM, the latter location being exposed to relatively high oxygen tensions. Lack of TCA cycle functions in the presence of oxygen has been explained by the ability of *Faecalibacterium* to use an extracellular electron shuttle of flavins and thiols, normally present in the healthy human gut, to transfer electrons to oxygen^34^. Further, our data confirm the major contribution of *Faecalibacterium* to butyrate production. GM-produced butyrate is beneficial for the gut epithelia as energy substrate, promotes mucous, antimicrobial peptides, and other components of the colonic defense barrier, modulates the host immune response, acting via receptor mediated or epigenetic mechanisms, stimulates secretion of gut hormones or directly targets organs and tissues, playing a role as signaling molecule in the human host, regulating hepatic and intestinal gluconeogenesis, and hepatic and fat tissues lipogenesis/lipolysis^35–38^. Instead, butyrate is not used as an energy source by gut bacteria. However, this specialized function of few Eubacteriales, mainly *Faecalibacterium*, might play a role also directly affecting the GM metabolism and composition. As reported recently, butyrate can cause cellular stress, membrane damage, and cell death in *Bacteroides*, effects that are species-dependent and conditional on which sugar is being utilized^39^. Hence, *Faecalibacterium* can be regarded as keystone taxon also for its direct effect on the GM, in addition to acting indirectly, as described above, by promoting a healthy mucosa.

Analysis of GM metabolic pathways was also focused on the relative contribution of GM members to benzoate metabolism. Benzoate is a metabolic end product of polyphenols, largely present in food. Furthermore, with the industrialization of food production, sodium benzoate is also largely used as a food preservative. Its antimicrobial activity on the GM has been shown to be negligible^40^. Accordingly, recent metagenomic analyses of human GMs from Europe, Asia and North America have highlighted the enrichment of benzoate catabolic features, associated with anaerobic (benzoyl-CoA degradation pathway) and aerobic (oxygenase-coupled central aromatic intermediate metabolic pathway) pathways, suggesting that its catabolism is a significant energy source for those bacteria which are equipped with the genes for benzoate degradation. However, functional assays with human fecal microbiota demonstrated that benzoate catabolites mapped only onto the aerobic oxygenase-coupled metabolism, showing lack of anaerobic degradation pathways^41^. Here, we provide direct evidence of the real abundance of benzoate catabolic functions expressed by the GM, as detectable in all datasets here evaluated. In contrast with the study of Yadav et al., metaproteomic data demonstrated that Bacillota provide the most relevant contribution to benzoate catabolism, including functions (benzoyl-CoA reductase and cyclohexanecarboxylate-CoA ligase) mapping into the anaerobic catabolic pathway of benzoate, while only aerobic features of the benzoate catabolism are assigned to Bacteroidota.

GM functions are of great interest also for investigating variations that might occur in aging. In this analysis, we did not observe correlations with age at any taxonomic or functional level (at least considering the most abundant features present in all datasets). Future studies with larger cohorts will be needed to further investigate the potential of metaproteomics in disentangling the link between GM and age.

This study has some relevant limitations that need to be pointed out. As mentioned above, the 10 datasets re-analyzed here were extremely heterogeneous in terms of sample preparation and analytical methods. This introduces relevant sources of non-biological variability, making it difficult to perform a robust and reliable comparison. Accordingly, we normalized abundance data and only considered mean values among all datasets, also providing rank-based results, with the aim of minimizing the influence of technical biases and batch effects and identifying the strongest trends (i.e., those consistently present in all datasets). Future multicentric studies analyzing samples from different cohorts worldwide (including developing countries) with standardized sample preparation and analytical protocols will be needed to confirm the findings of this study and to properly investigate differences between populations. Furthermore, state-of-the-art metaproteomics presents intrinsic limitations concerning data annotation in taxonomic and functional terms. Currently, lower taxonomic levels (i.e., genus or even species) can only be reached for a quite small percentage of the identified peptides (36% and 19% on average for genus and species levels, respectively, in this study), due to the presence of highly conserved sequences. In addition, although the percentage of functionally annotated peptides was rather high (around 75% on average in this study), some relevant functions could not be mapped to a KEGG pathway. This was the case of several (very abundant) proteins expressed by members of Bacteroidota and involved in polysaccharide transport and degradation, possibly explaining the unexpectedly low abundance of Bacteroidota starch metabolism within phylum-specific pathways. More generally, GM data are still strongly dependent on the databases used (and their update level) for taxonomic and functional annotation. Many new taxa have been introduced in the last years, making it challenging to compare data obtained even a few years ago with more recent results. Furthermore, KEGG functional annotation achieved using eggNOG-mapper ensures good annotation yields and interoperability between different levels, but shortcomings and issues clearly exist. In other words, a complete and unambiguous annotation is still a goal to be achieved. Finally, this study was focused on the bacterial and archaeal fractions of the fecal metaproteome, as the sample preparation protocols used to generate most of the datasets reanalyzed here (as well as to obtain the widely employed collection of human gut metagenomes used as sequence database for peptide identification) were not suited for the characterization of the less abundant fungal and viral fractions. Future investigations specifically aimed at analyzing the human fecal mycobiome and virome through metaproteomics will contribute to a deeper understanding of the complex interkingdom interactions occurring in the human colonic environment. At this regard, the information depth reached by metaproteomics is expected to increase considerably in the next years thanks to the latest advancements in mass spectrometry, enabling the detection of thousands of low-abundance proteins even in rather complex biological samples such as stool^42–44^.

In conclusion, this study allowed us to identify taxonomic and functional features consistently present with high abundance and frequency in the GM of healthy individuals from different countries, to detect co-occurrence and mutual exclusion dynamics involving bacterial taxa and functions, and to dissect the taxon-specific contribution to molecular functions, biological processes and metabolic pathways actively expressed by the healthy human GM, with a focus on SCFA biosynthesis, citrate cycle, flagellar assembly and quorum sensing. Our data provide a valuable contribution to the definition of the functional mechanisms regulating the relationship between the GM and the human physiology. These results encourage the use of fecal metaproteomics to investigate GM activity, opening the way to future multicentric studies with larger population cohorts and standardized analytical approaches.

## Methods

### Datasets

The datasets were selected based on the following inclusion criteria: human cohort including at least 5 healthy (clearly not labeled as diseased) adult ( > 18 years old) individuals; data derived from liquid chromatography (LC)-tandem mass spectrometry (MS) data-dependent label-free analysis of fecal samples (with neither subcellular fractionation of microbial cells nor offline fractionation of peptides); availability of raw MS data on public repositories. Eight published datasets fulfilled the inclusion criteria and were therefore included in the study.

Furthermore, we made available two unpublished datasets, also matching the inclusion criteria. The two datasets (labeled D08 and D09 in this study) were obtained from 12 and 10 healthy Sardinian subjects, respectively. All subjects gave their informed consent for using the biological material for research purposes and the studies were approved by the Ethics Committee of the University of Sassari, Italy (authorization no. 2023). In both cases, stool samples were stored at −80 °C within one hour after collection and then processed according to an established protocol, including bead beating in SDS-based buffer for protein extraction and a modified filter-aided sample preparation (FASP) for protein digestion^45^. LC-MS/MS analyses of the peptide mixtures were performed according to previous reports (ref. ^46^ for D08 and ref. ^47^ for D09), except for LC run duration (180 min for D08 and 310 min for D09).

As described in Table 1, the ten datasets re-analyzed in this study comprised a total of 134 MS raw files (each one coming from a different healthy human subject) and were highly heterogeneous in terms of sample preparation and mass spectrometry methods, as well as of geographic distribution.

### Bioinformatic analysis

Peptide identification was carried out using Proteome Discoverer™ (v.2.5; Thermo Fisher Scientific), with Sequest-HT as the search engine and Percolator for peptide validation, setting the false-discovery rate (FDR) threshold to 1%. The raw files of each dataset were re-analyzed in a separate batch. Search parameters were as follows: precursor mass range 350–5000 Da; minimum peak count 5; S/N Threshold 1.5, enzyme trypsin (full); maximum missed cleavage sites 2; peptide length range 6-50 amino acids; precursor mass tolerance 10 ppm; fragment mass tolerance 0.02 Da (0.5 Da for D04 and D05); static modification cysteine carbamidomethylation; dynamic modification methionine oxidation. Searches were conducted in parallel against two sequence databases, namely a collection of human gut metagenomes (available at https://ftp.cngb.org/pub/SciRAID/Microbiome/humanGut_9.9M/GeneCatalog/IGC.pep.gz)^3^ and the *Homo sapiens* protein sequences retrieved from Swiss-Prot (release 2019_08)^48^. Peptides were categorized as “microbial” or “human” when belonging to the first or second database, respectively.

Offline mass recalibration and label-free MS1 quantitation were performed using Spectrum Files RC and Minora Feature Detector nodes, respectively. Optimal settings for retention time and mass tolerance windows were calculated using the Minora algorithm based on the mass accuracy and retention time variance distribution. A consensus feature list was defined based on the outputs of Feature Mapper and Precursor Ions Quantifier nodes. The MS1 signals of all peptides significantly matching with at least one MS2 spectrum from at least one sample were mapped across runs and quantified by calculating the integrated area of the chromatographic peak^49^. The quantification pipeline was carried out separately for each dataset.

Unipept Desktop (v.2.0.0) was used to carry out peptide taxonomic annotation^50^, selecting the three available options (“equate I and L”, “filter duplicate peptides” and “advanced missed cleavage handling”). Protein sequences were subjected to functional annotation using the eggNOG-mapper web application (v.2.1.9, available at http://eggnog-mapper.embl.de/)^51^ keeping default parameters and then choosing KEGG (Kyoto Encyclopedia of Genes and Genomes) orthology (KO) information as the main functional classification^52^. Meta4P (v.1.4.4)^53^ was used to parse identification, quantification and annotation data and generate aggregated abundance tables for each dataset; only “microbial” peptides were selected for further analyses and their abundance data were re-normalized after filtering. The abundance of a taxon, a function, or a taxon-specific function was estimated by summing the abundance values associated with all peptides having that feature among their annotations. Features not assigned to bacterial or archaeal taxa were filtered out manually. The “map pathway” function on the KEGG website (http://www.kegg.jp) was used to map KO numbers on KEGG pathways.

### Statistical analysis and graph generation

Percentage mean abundance and coefficient of variation between subjects were calculated for each feature (taxa, functions and taxon-specific functions) in the single datasets; then, the values computed in the 10 datasets were averaged to obtain a global measure of abundance and variation for each feature. In parallel, all features were ranked based on their abundance in the metaproteome of each subject analyzed; then, median rank, interquartile range (IQR) and IQR/median ratio were calculated between the 134 subjects analyzed in the study.

Spearman’s correlation analysis was performed using two R packages: *mycor* for correlation between taxa (or functions) and subjects’ age, and *Hmisc* for correlation between taxa and taxa and between functions and functions. We calculated the Spearman’s coefficient of correlation (rho) between the most abundant features, considering the abundances measured for each dataset (separately). Then, we performed a random effect maximum likelihood (REML) meta-analysis (R package *metafor*) to obtain a combined estimate for each feature pair. Spearman’s correlation coefficients were Z-transformed before the meta-analysis using the Fischer Z transformation and then inverse-transformed after the meta-analysis, to avoid bias based on the expected non-normal distribution of Spearman’s estimates. The same approach was used for investigating the correlation between GM features and chronological age. Finally, we performed FDR correction for multiple testing on the nominal p-values according to the Benjamini-Hochberg (BH) method (significance threshold 0.05). The R package *corrplot* was to perform hierarchical clustering on correlation data and generate heatmaps.

For each microbial functional feature (KOs and pathways), the abundance values assigned the two main phyla (Bacillota and Bacteroidota) were subjected to logarithmic transformation and compared in all the subjects using a two-sided paired t test, followed by BH correction for multiple testing (significance threshold 0.05).

Histograms, violin plots, aligned dot plots and bar graphs were created with GraphPad Prism 9.

## Supplementary information

Supplementary information

Supplementary Data 1

Supplementary Data 2

Supplementary Data 3

Supplementary Data 4

Supplementary Data 5

## Data Availability

Mass spectrometry proteomics data have been deposited to the ProteomeXchange Consortium via the PRIDE^54^ partner repository with the dataset identifiers PXD046818.
