# Author Correction: Metaproteomic portrait of the healthy human gut microbiota

**DOI:** 10.1038/s41522-024-00635-0

**Published:** 2025-01-03

**Authors:** Alessandro Tanca, Antonio Palomba, Giovanni Fiorito, Marcello Abbondio, Daniela Pagnozzi, Sergio Uzzau

**Affiliations:** 1 Department of Biomedical Sciences, University of Sassari, Sassari, Italy; 2 Porto Conte Ricerche, Science and Technology Park of Sardinia, Tramariglio, Alghero Italy; 3 Clinical Bioinformatic Unit, IRCCS Istituto Giannina Gaslini, Genoa, Italy; 4 Unit of Microbiology and Virology, University Hospital of Sassari, Sassari, Italy

**Keywords:** Microbiome, Symbiosis

Correction to: *npj Biofilms and Microbiomes* 10.1038/s41522-024-00526-4, published online 28 June 2024

In the Fig. 4 caption beginning “Diameter and color of each circle (see legend on the right for color gradient) depend on the weighted average rho value computed via REML meta-analysis for that KO-KO correlation in the 10 datasets.” should have read “Diameter and color of each circle (see legend on the right for color gradient) depend on the weighted average rho value computed via REML meta-analysis for that pathway-pathway correlation in the 10 datasets.” Additionally few references were omitted from the paper which have now been added. The original article has been corrected.

