# Supplementary material for: Metaproteomic portrait of the healthy human gut microbiota
Source: NPJ Biofilms Microbiomes. 2024 Jun 28;10:54. doi: 10.1038/s41522-024-00526-4 (PMC11214629; doi:10.1038/s41522-024-00526-4)
Supplement: Supplementary file 1 — Supplementary information [file 41522_2024_526_MOESM1_ESM.pdf]

Supplementary Figures

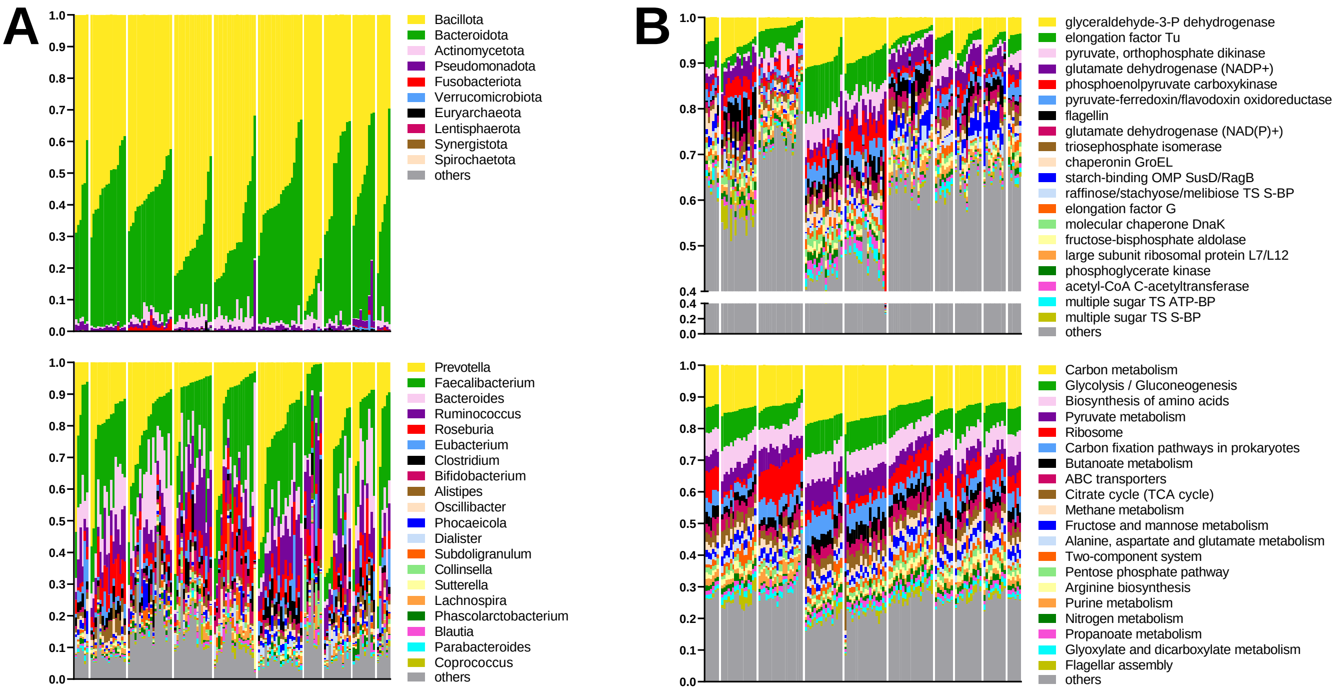

Supplementary Figure 1. Relative distribution of the main taxonomic and functional features among the subjects analyzed in the study. A blank vertical line separates data from different datasets (ordered from D01 to D10). A) Histograms showing the relative abundance distribution of top 10 microbial phyla (top) and top 20 microbial genera (bottom). B) Histograms showing the relative abundance distribution of top 20 KO functions (top) and KEGG pathways (bottom).

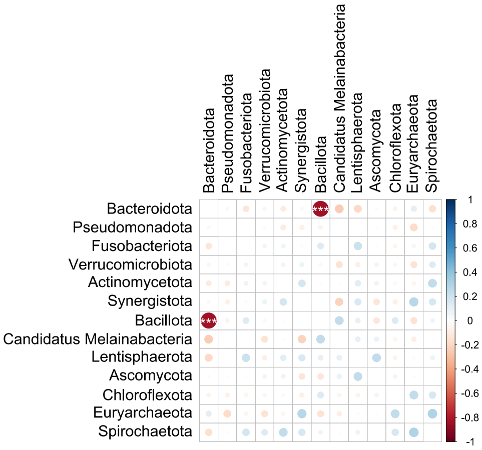

Supplementary Figure 2. Heatmap showing correlation trends between microbial phyla (those detected in at least 50% of subjects on average within the 10 datasets) according to Spearman's rho values. Phyla are ordered based on hierarchical clustering. Diameter and color of each circle (see legend on the right for color gradient) depend on the weighted average rho value computed via REML meta-analysis for that KO-KO correlation in the 10 datasets. Asterisks mark statistically significant correlations (*** = FDR<0.001; ** = FDR<0.01; * = FDR<0.05).

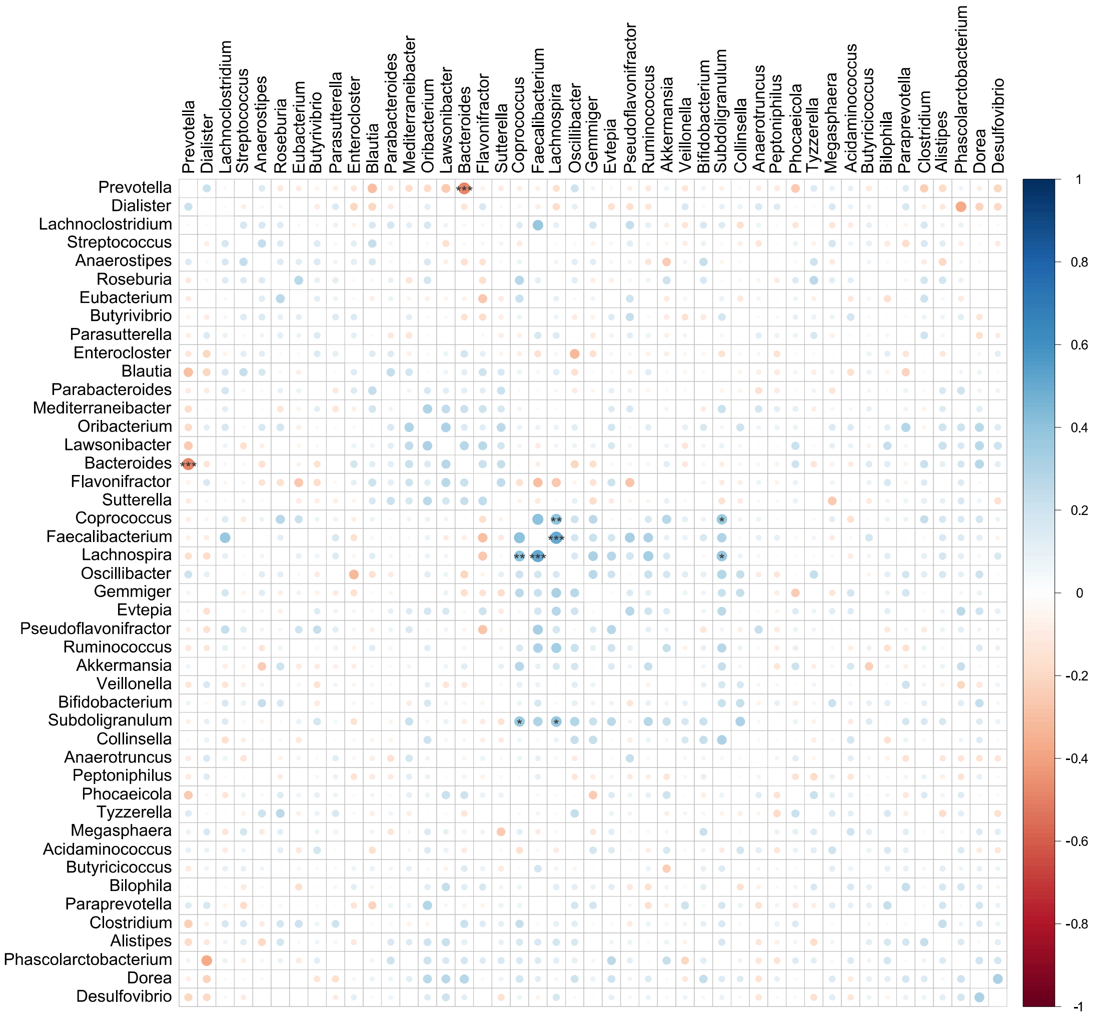

Supplementary Figure 3. Heatmap showing correlation trends between microbial genera (those detected in all datasets and in at least 75% of subjects on average) according to Spearman's rho values. Genera are ordered based on hierarchical clustering. Diameter and color of each circle (see legend on the right for color gradient) depend on the weighted average rho value computed via REML meta-analysis for that KO-KO correlation in the 10 datasets. Asterisks mark statistically significant correlations (*** = FDR<0.001; ** = FDR<0.01; * = FDR<0.05).

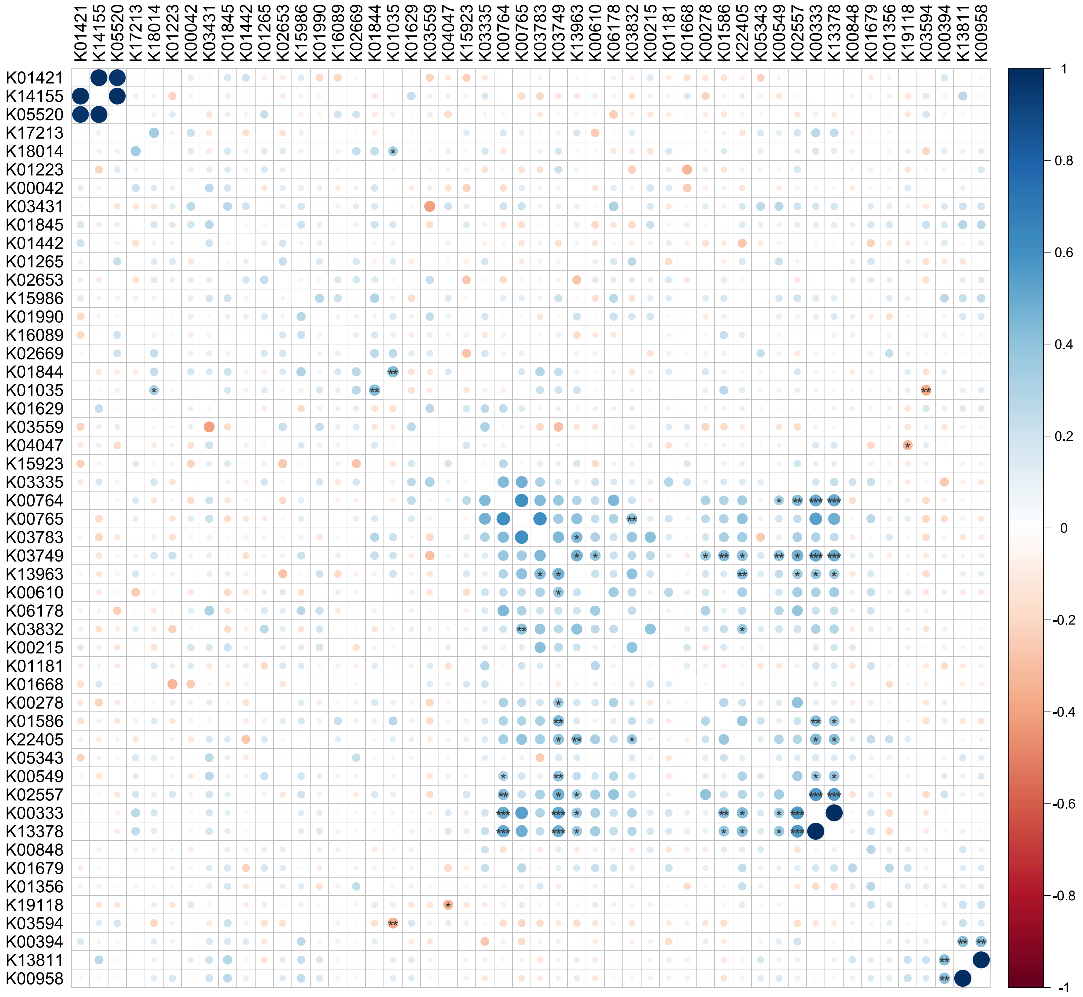

Supplementary Figure 4. Heatmap showing correlation trends (according to Spearman's rho values) between KO functions with the 50 highest mean coefficient of variation values, selected among those detected in at least 9 datasets. KO functions are ordered based on hierarchical clustering. Diameter and color of each circle (see legend on the right for color gradient) depend on the weighted average rho value computed via REML meta-analysis for that KO-KO correlation in the 10 datasets. Asterisks mark statistically significant correlations (*** = FDR<0.001; ** = FDR<0.01; * = FDR<0.05). K01421, putative membrane protein; K14155, cysteine-S-conjugate beta-lyase; K05520, protease I; K17213, inositol transport system substrate-binding protein; K18014, 3-aminobutyryl-CoA ammonia-lyase; K01223, 6-phospho-beta-glucosidase; K00042, 2-hydroxy-3-oxopropionate reductase; K03431, phosphoglucosamine mutase; K01845, glutamate-1-semialdehyde 2,1-aminomutase; K01442, choloylglycine hydrolase; K01265, methionyl aminopeptidase; K02653, type IV pilus assembly protein PilC; K15986, manganese-dependent inorganic pyrophosphatase; K01990, ABC-2 type transport system ATP-binding protein; K16089, outer membrane receptor for ferrienterochelin and colicins; K02669, twitching motility protein PilT; K01844, beta-lysine 5,6-aminomutase alpha subunit; K01035, acetate CoA/acetoacetate CoA-transferase beta subunit; K01629, rhamnulose-1-phosphate aldolase; K03559, biopolymer transport protein ExbD; K04047, starvation-inducible DNA-binding protein; K15923, alpha-L-fucosidase 2; K03335, inosose dehydratase; K00764, amidophosphoribosyltransferase; K00765, ATP phosphoribosyltransferase; K03783, purine-nucleoside phosphorylase; K03749, DedD protein; K13963, serpin B; K00610, aspartate carbamoyltransferase regulatory subunit; K06178, 23S rRNA pseudouridine2605 synthase; K03832, periplasmic protein TonB; K00215, 4-hydroxy-tetrahydrodipicolinate reductase; K01181, endo-1,4-beta-xylanase; K01668, tyrosine phenol-lyase; K00278, L-aspartate oxidase; K01586, diaminopimelate decarboxylase; K22405, NADH oxidase (H2O-forming); K05343, maltose alpha-D-glucosyltransferase / alpha-amylase; K00549, 5-methyltetrahydropteroyltriglutamate--homocysteine methyltransferase; K02557, chemotaxis protein MotB; K00333, NADH-quinone oxidoreductase subunit D; K13378, NADH-quinone oxidoreductase subunit C/D; K00848, rhamnulokinase; K01679, fumarate hydratase, class II; K01356, repressor LexA; K19118, CRISPR-associated protein Csd2; K03594, bacterioferritin; K00394, adenylylsulfate reductase, subunit A; K13811, 3'-phosphoadenosine 5'-phosphosulfate synthase; K00958, sulfate adenylyltransferase.

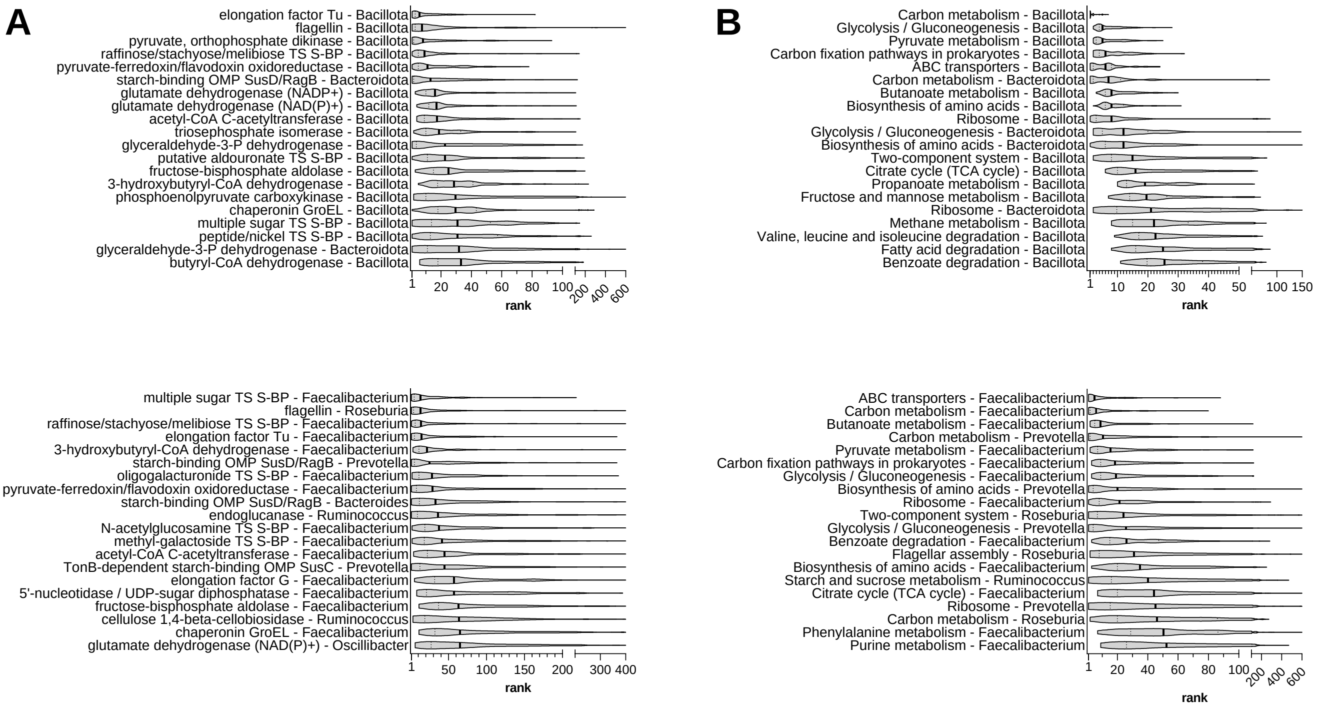

Supplementary Figure 5. Main taxon-specific functional features of the healthy human gut metaproteome. Violin plots were based on the distribution of rank data, as observed in the 134 subjects analyzed in this study. A) Top 20 phylum-specific (top) and genus-specific (bottom) KO functions, ordered by decreasing median rank. TS, transport system; S-BP, substrate-binding protein; OMP, outer membrane protein. B) Top 20 phylum-specific (top) and genus-specific (bottom) KEGG pathways, ordered by decreasing median rank.

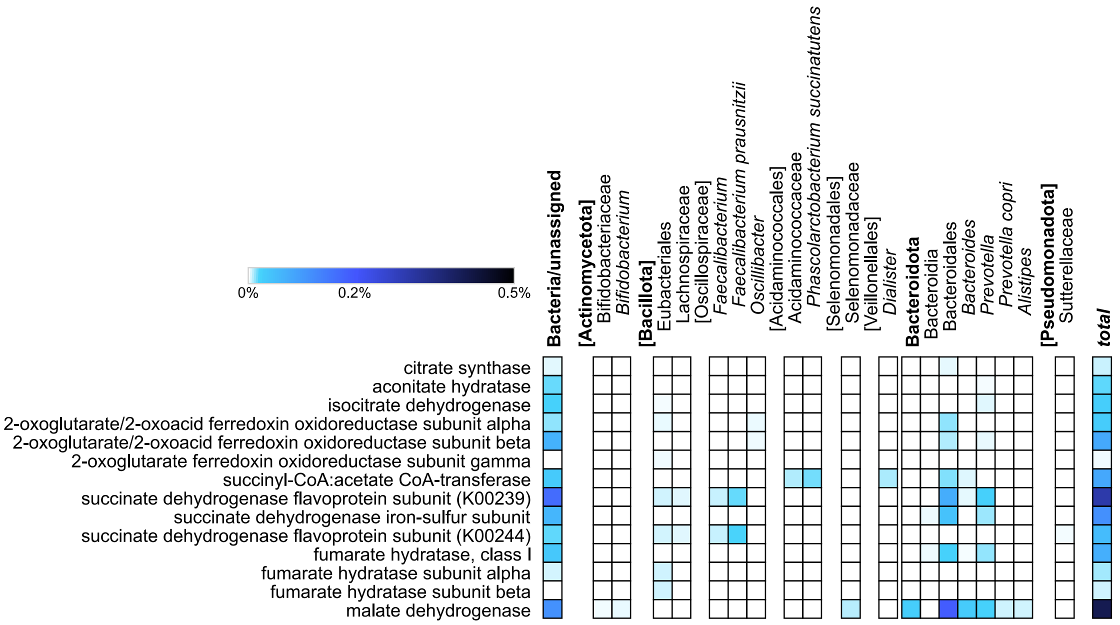

Supplementary Figure 6. Taxon-specific distribution of enzymes involved in the "Citrate cycle (TCA cycle)" KEGG pathway. KO functions detected in more than 6 datasets with a mean abundance higher than 0.001% are shown. The color of each square corresponds to the mean percentage abundance between the 10 datasets (see color legend). Phyla are in bold, genera and species in italic, while higher taxa useful for classification but unassigned to any of the listed enzymes are into square brackets. The column "total" corresponds to the summed abundance of all taxon-specific assignments (including "Bacteria/unassigned") for a given enzyme

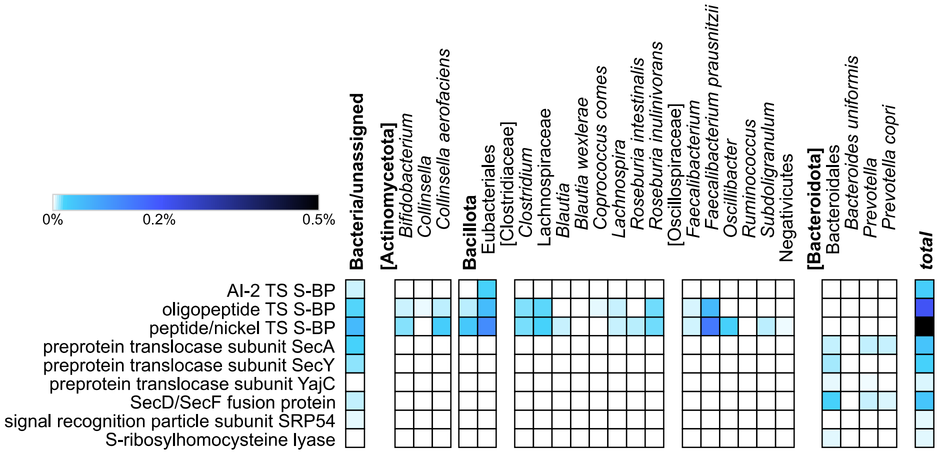

Supplementary Figure 7. Taxon-specific distribution of functions involved in the "Quorum sensing" KEGG pathway. KO functions detected in more than 6 datasets with a mean abundance higher than 0.001% are shown. The color of each square corresponds to the mean percentage abundance between the 10 datasets (see color legend). Phyla are in bold, genera and species in italic, while higher taxa useful for classification but unassigned to any of the listed proteins are into square brackets. The column "total" corresponds to the summed abundance of all taxon-specific assignments (including "Bacteria/unassigned") for a given enzyme. TS, transport system; S-BP, substrate-binding protein.

Supplementary Data legends

Supplementary Data 1. Summary tables reporting the number of quantified peptides for each annotation category, for each sample and for the whole dataset, along with the number of quantified features for each annotation level (taxa, functions and taxon-specific functions).

Supplementary Data 2. Phyla, genera, KO functions, KEGG pathways, plus taxon-specific KOs and pathways, identified in the 10 datasets re-analyzed in this study. Relative abundance, coefficient of variation, subjects and number of datasets in which the feature was detected are provided for each feature as mean values (both between datasets and between subjects of the same dataset), along with values measured for each subject.

Supplementary Data 3. Phyla, genera, KO functions, pathways, taxon-specific KOs and pathways identified in the 10 datasets re-analyzed in this study. Median rank, interquartile range (IQR) and IQR/median ratio between the 134 subjects analyzed in the study are provided for each feature, along with rank values measured for each subject.

Supplementary Data 4. Correlation analysis results. Spearman's rho (weighted mean with lower and upper confidence interval bounds), p- and FDR values computed via REML meta-analysis are provided for correlations between phyla (those detected in at least 50% of subjects on average within the 10 datasets), genera (those detected in all datasets and in at least 75% of subjects on average), KO functions (top 50 of those detected in all datasets and in at least 75% of subjects on average; top 50 of those detected in at least 9 datasets with the highest mean coefficient of variation) and pathways (those detected in all subjects, with minimum 1% of mean abundance), as well as for correlations between subjects' age and phyla, genera, KO and pathways.

Supplementary Data 5. Taxon-specific KO functions and pathways. Lists of KO functions and pathways with significantly differential abundance in the Bacillota vs Bacteroidota comparison, along with abundance log ratios, p-values and q-values (FDR) obtained upon application of paired t test, are shown. Two further sheets provide lists of genus-specific KO functions, selected among those identified in ≥50% of subjects and with <50% missing assignments at the genus level, respectively those exclusive to a genus (i.e., assigned at ≥90% to a single genus) and those shared between two or more genera (i.e., not assigned at ≥75% to a single genus).
